# In Memoriam: Dr Dan Joseph Stein (1962–2025)

**DOI:** 10.1002/gps3.70005

**Published:** 2026-03-11

**Authors:** Jinghong Chen, Liya Sun, Min Zhao, Zhen Wang

**Affiliations:** ^1^ Shanghai Mental Health Center Shanghai Jiao Tong University School of Medicine Shanghai China

What we have done for ourselves alone dies with us; what we have done for others and the world remains and is immortal.

—Albert Pike

With profound sorrow, *General Psychiatry* commemorates the life and legacy of Dr Dan Joseph Stein, a South African psychiatrist and neuroscientist whose work had a lasting influence on contemporary psychiatry across continents and disciplines.

Dr Stein passed away in December 2025 at the age of 63. At the time of his passing, he served as professor and chair of the Department of Psychiatry and Mental Health at the University of Cape Town and held senior leadership roles within the South African Medical Research Council, directing research programmes focused on mental disorders, psychological risk and resilience,^1^ and obsessive‐compulsive disorder (OCD).

Throughout his career, Dr Stein articulated and consistently pursued an integrated scientific vision that linked basic neuroscience, clinical psychiatry and population‐based mental health research, transcending disciplinary boundaries between psychiatry, psychology and philosophy.^2^, ^3^ His work consistently reflected a conviction that scientific excellence should translate into meaningful improvements in mental health care, policy and societal wellbeing.^2^, ^3^

Beyond individual achievements, Dr Stein was widely recognised as a builder of academic communities, fostering collaboration across disciplines and institutions while maintaining high intellectual standards. Dr Stein was a longtime friend of the Shanghai Mental Health Center (SMHC). Through joint research initiatives, scholarly dialogue and sustained academic exchange, he contributed to strengthening connections between Chinese psychiatry and the broader international community, particularly at the intersection of neuroscience, psychiatric classification and global mental health and produced a significant body of high‐quality research, especially on OCD,^4^ the International Classification of Diseases, 11th Revision (ICD‐11),^5^ World Health Organization (WHO) surveys^6^ and the Global Burden of Disease study.^7^ In 2017, Dr Stein visited SMHC and participated in the WHO ICD‐10 Mental and Behavioural Disorders Revision Field Studies Coordination and International Advisory Group Meeting, as well as the WHO Global Mental Health Shanghai Forum. His keynote speech ‘*Behavioural addictions: where next*?’ made significant contributions to the development of ICD‐11 diagnostic guidelines and global mental health collaboration. He later continued this engagement for SMHC at the Second Oriental Congress of Psychiatry Forum in September 2025, taking part in high‐level discussions on the future of psychiatry. He also nurtured a long‐term academic relationship with Professor Zhen Wang's group at SMHC, serving as an indispensable counsellor for local OCD projects such as PROCEED.^8^ One of the papers he coauthored has been published in *Nature Mental Health* this year, presenting a new treatment strategy for OCD.^9^

Dr Stein was a deeply valued member of the *General Psychiatry* Editorial Board, serving the journal with generosity and dedication as both a reviewer and an academic advisor. He offered not only rigorous scholarly judgement and methodological insight but also a rare breadth of strategic vision, helping to shape and strengthen the journal's mission to advance high‐quality, internationally relevant psychiatric research.

**FIGURE 1:**
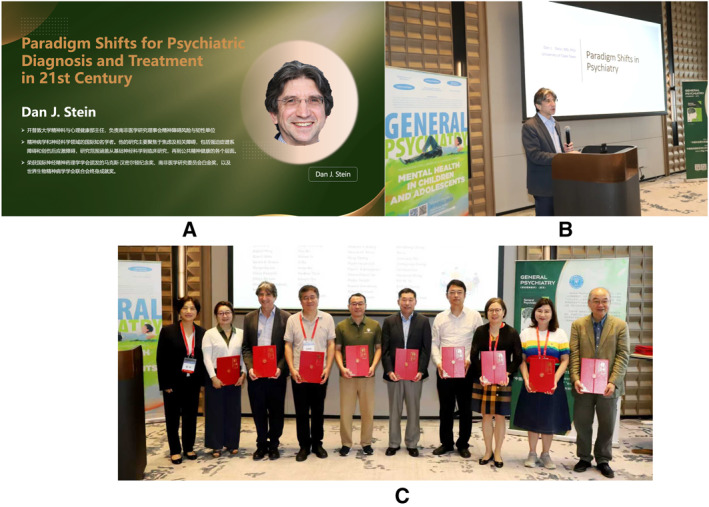
Photos from *General Psychiatry* 2025 Editorial Board Meeting. (A) The introduction slide that Dr Stein personally expressed appreciation for. (B) Dr Stein delivering a keynote speech. (C) Editorial Board appointment ceremony.

In September 2025, he attended the *General Psychiatry* Editorial Board meeting in person and delivered a keynote lecture entitled ‘*Paradigm shifts in psychiatry*’ (figure 1). His reflections on strict scientism (evidence‐based) versus soft naturalism (values‐based)^10^ and advocacy for the precise advancement of psychiatry by incremental integration of a range of valid models^11^ were widely appreciated for their intellectual depth, clarity and forward‐looking perspective and left a lasting impression on all who were present. Beyond his formal roles in the Editorial Board, Dr Stein was unfailingly generous with his time and wisdom. He offered thoughtful professional guidance and warm encouragement to our editorial team, engaging in discussions on the journal's international presence and long‐term development. His involvement was marked by a profound respect for academic standards, as well as a spirit of collegiality and kindness that will be long remembered. We had invited him to contribute a review on the current status and future of adolescent mental health in Africa for our journal, an opportunity we deeply valued that was not to be.

In remembering Dr Stein, we honour not only an extraordinary body of scientific work, but also the person behind it—a scholar who was exacting in his intellectual standards yet unfailingly generous in collaboration, global in outlook yet deeply attentive to individual human suffering. He combined rigour with kindness and vision with humility. His presence, guidance and friendship will be deeply missed and his legacy will continue to inspire the global psychiatric community.

Such a One is never dead—His heart, his mind, his soul: not dead.

—Douglas Livingstone
